# Health economics of rubella: a systematic review to assess the value of rubella vaccination

**DOI:** 10.1186/1471-2458-13-406

**Published:** 2013-04-29

**Authors:** Joseph B Babigumira, Ian Morgan, Ann Levin

**Affiliations:** 1 Global Medicines Program, Department of Global Health, University of Washington, Seattle, WA, USA; 2 Pharmaceutical Outcomes Research and Policy Program, Department of Pharmacy, University of Washington, Seattle, WA, USA; 3 St. Mary’s College of Maryland, Lexington Park, MD, 20653, USA; 4 Independent consultant, Bethesda, MD, USA

## Abstract

**Background:**

Most cases of rubella and congenital rubella syndrome (CRS) occur in low- and middle-income countries. The World Health Organization (WHO) has recently recommended that countries accelerate the uptake of rubella vaccination and the GAVI Alliance is now supporting large scale measles-rubella vaccination campaigns. We performed a review of health economic evaluations of rubella and CRS to identify gaps in the evidence base and suggest possible areas of future research to support the planned global expansion of rubella vaccination and efforts towards potential rubella elimination and eradication.

**Methods:**

We performed a systematic search of on-line databases and identified articles published between 1970 and 2012 on costs of rubella and CRS treatment and the costs, cost-effectiveness or cost-benefit of rubella vaccination. We reviewed the studies and categorized them by the income level of the countries in which they were performed, study design, and research question answered. We analyzed their methodology, data sources, and other details. We used these data to identify gaps in the evidence and to suggest possible future areas of scientific study.

**Results:**

We identified 27 studies: 11 cost analyses, 11 cost-benefit analyses, 4 cost-effectiveness analyses, and 1 cost-utility analysis. Of these, 20 studies were conducted in high-income countries, 5 in upper-middle income countries and two in lower-middle income countries. We did not find any studies conducted in low-income countries. CRS was estimated to cost (in 2012 US$) between $4,200 and $57,000 per case annually in middle-income countries and up to $140,000 over a lifetime in high-income countries. Rubella vaccination programs, including the vaccination of health workers, children, and women had favorable cost-effectiveness, cost-utility, or cost-benefit ratios in high- and middle-income countries.

**Conclusions:**

Treatment of CRS is costly and rubella vaccination programs are highly cost-effective. However, in order for research to support the global expansion of rubella vaccination and the drive towards rubella elimination and eradication, additional studies are required in low-income countries, to tackle methodological limitations, and to determine the most cost-effective programmatic strategies for increased rubella vaccine coverage.

## Background

Acquired rubella is a mild disease which only rarely results in serious clinical complications i.e. encephalitis, arthritis and thrombocytopenia. The mild nature of the disease means that vaccination against it would not be medically or economically critical or justifiable if it did not infect pregnant mothers. During pregnancy, particularly in the first trimester, rubella infection is far more dangerous and commonly leads to abortion, fetal death or congenital rubella syndrome (CRS) [1]. CRS, which is estimated to affect 110,000 infants annually in low-income countries [2], is characterized by multiple defects of the brain, heart, eyes and ears and is an important cause of hearing and visual impairment and mental retardation. CRS often causes lifelong physical and mental disability, requiring costly institutional care and special schools, and using a large amount of healthcare and societal resources. Therefore CRS provides the medical and economic rationale for appropriate prophylaxis with a vaccine.

A live attenuated vaccine against rubella has been available for over 40 years. It has a high immunogenicity, produces sero-conversion in close to 100% of vaccines, and confers immunity similar to that of natural infection [1]. Immunity is maintained for at least 20 years and although infection, including CRS, may occur in vaccinated individuals with low antibody titers, this is not an important factor in rubella epidemiology [1]. The rubella vaccine is available in combination with measles (MR) and with measles and mumps (MMR) as well as in monovalent form (R). Combination with other vaccines does not affect immunogenicity [1].

Most high- and middle-income countries include rubella-containing vaccine (RCV) in their childhood immunization schedules and have made progress in reducing the incidence of rubella or eliminating it. The World Health Organization (WHO) Latin American region (AMR) has eliminated rubella and CRS [3] and the European region (EUR) registered a 98% reduction in cases between 2000 and 2009 [4].

Although WHO published a position paper to guide introduction of RCV into the national childhood immunization schedules of member countries in 2000 [5], only a few low-income countries have included the vaccine in their schedules [4]. This is reflected in the epidemiological trends: the WHO eastern Mediterranean region (EMR) registered only a 35% reduction in rubella cases between 2000 and 2009 and the WHO African region (AFR) and South East Asian region (SEAR) registered 20-fold and 14-fold increases in rubella cases respectively during the same time period [4]. Of the 165 reported cases of CRS in 2009, AFR, with 47, had the highest number [4].

Low-income countries have been slow to add RCV to their national immunization schedules because of several reasons, two of which stand out. First, despite its relatively low price (as might be supplied to UNICEF or through a GAVI Alliance subsidy), adding rubella to the vaccine schedule might be prohibitively costly in countries where healthcare budgets are already stretched. This suggests that including RCV in immunization schedules in poor countries is not cost-effective compared to other public health interventions. Second, because rubella causes a substantial proportion of its damage in fetuses, a high level of vaccination coverage (over 80%) must be maintained to avoid the risk of increasing the incidence of CRS which would happen if poor vaccine coverage reduced viral circulation in the population enough to shift rubella susceptibility from children to young mothers. This creates a unique policy problem: including RCV in national immunization schedules is likely not enough; the rubella immunization programs need to achieve herd immunity and many countries are unable to feasibly sustain such coverage standards.

A more recent position paper by WHO recommended that countries leverage accelerated measles control and elimination activities to introduce RCV and exploit this synergy to advance rubella and CRS elimination [6]. In line with the WHO recommendation, the GAVI Alliance is supporting large-scale catch up measles-rubella campaigns with the aim of reaching over 700 million children in 49 countries by 2020 [7].

Health economics, the study of how scarce healthcare resources are deployed and should be allocated in healthcare systems, has gained increasing prominence globally in the face of slowing western economies and continued resource constraints in low-income countries. Donors are increasingly scrutinizing the use of resources to assist poor countries in fighting disease and governments in low-income countries are increasingly scrutinizing their healthcare expenditures. Economic evaluations help policy makers to make resource allocation decisions by identifying different policy strategies, transparently evaluating their costs and benefits, quantifying the uncertainty around estimates, and examining different scenarios.

In 2002, Hinman et al. performed a global review of economic analyses of rubella and rubella vaccines published between 1970 and 2000 [8]. They found that inclusion of rubella vaccination in national immunization programs is both cost-beneficial and cost-effective and recommended further studies using data from the burden of rubella in developing countries and standardized methodologies [8].

In this paper, we present findings of an updated review of economic analyses of rubella and rubella vaccination. We examine the evidence on costs of rubella and CRS, the cost-effectiveness of adding RCV to national immunization programs, and the cost-effectiveness of different policy strategies that might be employed to add RCV to national childhood immunization schedules. Our aim is to examine the economic evidence base, assess differences in findings by country income levels, identify gaps in the evidence, and propose potential areas of future enquiry into the economics of rubella and rubella vaccination. Our findings will support the planned global expansion of RCV and the push towards potential rubella elimination and eradication.

## Methods

We reviewed studies published in English on the costs and resource use for rubella and CRS and the costs, cost-effectiveness or cost-benefit of rubella vaccination between 1970 and 2012. This review is up-to-date as of December 1^st^ 2012 and conforms to the Preferred Reporting Items for Systematic Reviews and Meta-Analyses (PRISMA) guidelines [9].

We performed a systematic search of MEDLINE (PubMed) and the National Health Services Economic Evaluation Database. Our search strategy included the following terms: ‘rubella and economics’, ‘rubella and costs’, ‘rubella and cost-effectiveness’, ‘rubella and cost-utility’, ‘rubella and cost-benefit’, ‘CRS and economics’, and ‘CRS and costs’. The search limits were set to the custom range of dates as described above. The studies identified were reviewed one-by-one by reading their abstracts and identifying the design of the study as reported. The review included health economic evaluations i.e. cost analyses, cost-effectiveness analyses, cost-utility analyses, cost-benefit analyses, cost-consequences analyses, and cost-minimization analyses. The economic evaluation studies were further reviewed to identify any references that did not show up in the initial search.

After reviewing the studies chosen, we categorized them by study design and income level of countries in which they were performed. We used the World Bank definition, which categorizes countries according to Gross National Income (GNI) per capita in 2011 as follows: low income, $1,025 or less; lower middle income, $1,026 - $4,035; upper middle income, $4,036 - $12,475; and high income, $12,476 or more [10]. For the cost-effectiveness and cost-utility analyses, we used the 16-item Quality of Health Economics Studies (QHES) questionnaire [11] to assess study quality. This validated instrument can be used to quickly and accurately measure aspects of the quality of health economics studies on a scale of 0 – 100 and has been used to identify evidence to enhance decision making [12]. A higher score on the QHES indicates a study of better quality. We used the results to identify potential gaps in the evidence on the economics of CRS and rubella vaccination as it relates to the planned expansion of RCV and the potential for the global interruption of rubella transmission.

## Results

Our initial search identified 976 studies whose abstracts were reviewed and we selected 27 relevant studies on the economics of rubella, CRS, and rubella vaccination: 11 cost analyses, 11 cost-benefit analyses, 4 cost-effectiveness analyses, and 1 cost-utility analysis.

We included studies of the costs of CRS, cost analyses comparing strategies to screen healthcare workers and identify candidates for immunization, cost analyses comparing vaccination programs, and full economic evaluations of healthcare programs.

### Study design

Of the 11 cost analyses selected, 4 examined the costs of CRS [13-16], 4 compared the costs of blind and targeted vaccination of health care workers [17-20], 1 compared the costs of fully immunizing a child using different vaccines [21], 1 compared different approaches to vaccinating children, adolescent girls, and women [22], and one compared different strategies for testing pregnant women followed by immunization of susceptible women after birth [23].

All 11 cost-benefit analyses [24-34] compared different programmatic approaches to vaccination in the general population targeting children, girls, or women and 2 of the studies evaluated programs aimed at rubella eradication.

All 4 cost-effectiveness analyses [35-38] and the single cost-utility analysis [39] compared different programmatic approaches to vaccination in the general population and 1 study evaluated a program aimed at rubella eradication [36]. Of the 5 cost-effectiveness and cost-utility analyses, we had enough information to score 3 studies; one study was based on summary results in a report [36] and the other was reported in Slovak [37]. The three studies scored 30 [35], 62 [39] and 93 [38] on the QHES (on a scale of 0 – 100).

Twenty studies were conducted in high-income countries, 5 in upper-middle-income countries and two in lower-middle-income countries. We did not find any studies conducted in low-income countries.

### Cost analyses

#### Studies of the costs of congenital rubella syndrome (CRS)

All costs were converted to 2012 US dollars for comparison using historical consumer price indices for all countries based on data from the International Monetary Fund [40]. Table 1 is a summary of the four published studies of the cost of congenital rubella syndrome (CRS), three of which were from upper-middle-income countries. Three studies [14-16] evaluated the annual direct costs of treatment of CRS (hospitalization, outpatient visits, diagnostic tests, surgery, drugs and equipment) which ranged from (2012) $4,261 in Brazil [14] to (2012) $57,010 in Jamaica [16]. Differences were found among the studies with regard to the goods and services that were included in the cost analyses, e.g., some studies included the costs of surgery and cochlear implants while others did not (Table 1). The fourth study [13] estimated the lifetime direct costs of treatment and indirect productivity losses due to CRS in Oman to be (2012) $139,910.

**Table 1:** Studies of the cost of congenital rubella syndrome

**First author [Reference]**	**de Owens [**[15]**]**	**Robinson [**[16]**]**	**Lanzieri [**[14]**]**	**Al-Awaidy [**[13]**]**
Country	Panama	Jamaica	Brazil	Oman
Year	1989	1998	2004	2006
WB income group	Upper middle	Upper middle	Upper middle	High
Perspective	Health system*	Health system*	Health system	Societal
Cost components measured	NR	NR	Diagnosis; OP care; Hospitalization Surgery; 1^st^ year of FU	Diagnosis; OP care; Hospitalization Surgery; Drugs; Equipment; Special schools; Indirect costs
Method of cost estimation	NR	NR	Micro-costing using reimbursement data	Micro-costing using accounts data (treatment costs); Human capital approach (for indirect costs)
Time period for costing	Annual	Annual	Annual (1^st^ year)	Lifetime
Discounting (Rate)	NA	NA	NA	Yes (3%)
Results (2012 US$)	$58,023	$57,010	$4,261	$139,910
Sponsor	NR	NR	NR	Oman MOH*

*Not explicitly reported but inferred.

*WB*, World Bank; *NR*, Not Reported; *NA*, Not Applicable; *OP*, Out-Patient; *FU*, Follow-Up; *CRS*, Congenital Rubella Syndrome; *MOH*, Ministry of Health.

#### Cost analyses of vaccination programs for health care workers

Healthcare workers without a history of acquired rubella are susceptible to infection in health facilities. Screening detects rubella antibodies in serum using serological tests and screening programs are designed to identify individuals without antibodies, who are candidates for immunization. Analysts have compared the costs of different strategies to identify and immunize susceptible health workers. Table 2 is a summary of the four published cost analyses that evaluated the costs of providing rubella containing vaccines to health care workers. Two strategies were compared: vaccinating all health care workers (blind vaccination) vs. screening and vaccinating only the health workers whose tests were negative for rubella antibodies. Two of the studies took place in high-income countries [17,18] while the other two [19,20] were performed in Turkey, an upper- middle-income country. The two studies in high-income countries found that the costs of screening and vaccinating were lower and that this was preferable to blind vaccination, although Ferson et al. [18] found that blind vaccination might be preferable when conditions made testing an administrative burden. The findings of the two studies from Turkey were mixed: one [19] found that the costs of blind vaccination were slightly higher than targeting and the other [20] found that blind vaccination was less costly.

**Table 2:** Cost analyses of vaccination programs for healthcare workers

**First author [Reference]**	**Stover [**[17]**]**	**Ferson [**[18]**]**	**Celikbas [**[19]**]**	**Alp [**[20]**]**
Country	USA	Australia	Turkey	Turkey
Year	1994	1994	2006	2012
WB income group	High	High	Upper middle	Upper middle
Comparators	1. Screen & vaccinate	1. Vaccinate all	1. Screen & vaccinate	1. Screen & vaccinate
	2. Blind vaccination	2. Vaccinate if no disease history	2. Blind vaccination	2. Blind vaccination
		3. Test if no disease history then vaccinate		
		4. Test all and vaccinate		
Perspective	Payer*	Payer*	Payer*	Payer*
Cost components measured	Vaccine; laboratory; employee health services	Vaccine; venipuncture; laboratory consumables; personnel (serology)	Vaccine; serology	Vaccine; serology
Method of cost estimation	Micro-costing	Micro-costing	Micro-costing	Micro-costing
Time period for costing	One-time vaccination	One-time vaccination	One-time vaccination	One-time vaccination
Discounting (Rate)	NA	NA	NA	NA
Results (2012 US$)	1. $24	1. $5 – $37	1. $14	1. $13
	2. $71	2. $5 – $28	2. $18	2. $9
		3. $5 – $28		
		4. $9 - $42		
Stated conclusion	Screen and vaccinate preferable	A combination if screening and history is preferable	Blind vaccination modestly increased costs	Blind vaccination was preferable
Sponsor	NR	NR	TSRC	None

*Not explicitly reported but inferred.

*WB*, World Bank; *NR*, Not Reported; *NA*, Not Applicable; *TSRC*, Turkish Science Research Council.

#### Cost analyses of rubella vaccination programs

Three analyses from high-income countries evaluated the costs of various strategies to introduce rubella vaccine (Table 3). Gudnadottir [22] compared four strategic programmatic approaches in Iceland to preventing CRS by routine immunization using the monovalent Rubella RA27/3 vaccine: 1) the United States program at the time (vaccination of all children to create herd immunity and later (when CRS did not disappear), revaccinating all young adult women); 2) the United Kingdom program at the time (vaccination of teenage girls and sero-negative women (plus contraception for three months after vaccination)); 3) the Sweden program at the time (vaccination in early childhood and re-vaccination of all teenagers (general re-vaccination)) and 4) selective vaccination involving puerperal women as well as all women of child bearing age who are willing to use contraceptives for three months. The study found that systematic screening of women and teenage girls combined with vaccination of sero-negative individuals was the most cost-effective strategy for reduction of CRS in Iceland, followed by vaccination of 12 – 13 year old school girls.

**Table 3:** Cost analyses of vaccination programs in the general population

**First author [Reference]**	**Gudnadottir [**[22]**]**	**Fontanesi [**[21]**]**	**Haas [**[23]**]**
Country	Iceland	USA	USA
Year	1985	2004	2005
WB income group	High	High	High
Group targeted	Children	Children	Pregnant women
Comparators	1. Vaccinate all children and re-vaccinate women	1. MMR vaccination	1. Rubella test and rubella vaccine
	2. Vaccinate girls and sero-negative women	2. DTaP vaccination	2. Rubella test and MMR vaccine
	3. Vaccinate all children and re-vaccinate girls	3. IPV vaccination	3. MMR test and MMR vaccine
	4. Vaccinate screened women		4. MMR test and rubella/MMR vaccine
Perspective	Payer*	Payer*	Payer*
Cost components measured	Vaccine; serology	Vaccine; personnel; syringes	Vaccine; serology
Method of cost estimation	Micro-costing	Micro-costing	Micro-costing
Time period for costing	One-time vaccination at different ages	One-time vaccination (complete number of doses)	One-time vaccination
Discounting (Rate)	NA	NA	NA
Results (2012 US$)	1. $1,063	1. $30	1. $8
	2. $638	2. $23	2. $10
	3. $2,409	3. $18	3. $37
	4. $283		4. $35
Stated conclusion	Vaccination of girls and of women after screening was preferable	The MMR vaccine had the highest cost per immunized child	The combined MMR test and vaccination was the most costly
Sponsor	Iceland MOHSS	US CDC	NMBS

*Not explicitly reported but inferred.

*WB*, World Bank; *NR*, Not Reported; *NA*, Not Applicable; *MOHSS*, Ministry of Health and Social Services; *MMR*, Measles Mumps Rubella; *DTaP*, Diptheria Mumps Rubella; *IPV*, Inactivated Polio Vaccine; *CDC*, Centers for Disease Control; *NMBS*, Navy Bureau of Medicine and Surgery.

Fontanesi et al. [21] compared the costs of fully immunizing a child using MMR, diphtheria–tetanus–attenuated pertussis (DTaP) vaccine, and the inactivated polio vaccine (IPV) in the US. They estimated that the average cost of providing a single MMR vaccination (in 2012 US $) was US$30 compared to $23 for DTaP and $18 for IPV. Haas et al. [23] compared various strategies for vaccination of pregnant women: 1) rubella testing and rubella vaccination, 2) rubella testing and MMR vaccination, 3) MMR testing and MMR vaccination, and 4) MMR testing and rubella only or MMR vaccination. As expected, it was less costly to screen for rubella than for MMR.

#### Cost-benefit analyses of rubella vaccination programs

Tables 4, 5, and 6 present summaries of the eleven cost-benefit studies of rubella vaccination. Of these, ten studies were performed in high-income countries^1^, while the other (Irons et al. [34]) was performed in a number of upper-middle-income countries. The studies compared the costs and benefits of 1) vaccinating with MMR vs. monovalent rubella vaccine, 2) vaccinating various age groups vs. no program, 3) introducing a second dose of MMR, and 4) rubella elimination.

**Table 4:** Cost-benefit analyses of vaccination programs in the general population

**First author [Reference]**	**Stray-Pedersen [**[25]**]**	**White [**[26]**]**	**Hatzandrieu [**[27]**]**	**Schoenbaum [**[24]**]**
Country	Norway	USA	USA	USA
Year	1982	1985	1994	1976
WB income group	High	High	High	High
Comparators	1. Vaccinate infant girls	1. Rubella vaccination	1. Rubella vaccination	1. Vaccinate all 2-yr-olds
	2. Vaccinate pubertal girls	2. MMR vaccination	2. MMR vaccination	2. Vaccinate all 6-yr-olds
				3. Vaccinate all 12-yr-olds
				4. Vaccinate 2-yr-olds and 12-yr-olds
Perspective	Societal	Societal	Societal	Societal
Cost components measured	Vaccine; immunization; serology; CRS treatment (including special care; indirect costs (lost productivity and premature mortality)	Vaccine; immunization; physician visits; hospitalization; supportive care; special schooling; institutionalization; indirect costs (lost wages, lost lifetime earnings due to retardation or death)	Vaccine; immunization; physician visits; hospitalization; supportive care; special schooling; institutionalization; indirect costs (lost wages, lost lifetime earnings due to retardation or death)	Vaccine; immunization; OP care; hospitalization; CRS treatment and care; indirect costs (lost lifetime earnings)
Method of cost estimation	Micro-costing (for vaccination and treatment; expected lifetime earnings (for indirect costs)	Micro-costing (for direct costs; expected lifetime earnings (for indirect costs)	Micro-costing (for direct costs; expected lifetime earnings (for indirect costs)	Micro-costing (for direct costs; expected lifetime earnings (for indirect costs)
Method of benefits estimation	Averted costs	Averted costs	Averted costs	Averted costs
Time period for costs and benefits	Lifetime	Lifetime	Lifetime	Lifetime
Discounting (Rate)	Yes (7%)	Yes (10%)	Yes (10%)	Yes (6%)
Results—Benefit-cost ratio	1. 5	1. 7.7	1. 11.1	1. 8
	2. 11	2. 14.4	2. 21.3	2. 9
				3. 27
				4. 8
Stated conclusion	Vaccination of pubertal girls preferable	Routine MMR vaccine program was cost-effective	Routine MMR vaccine program was cost-effective	Vaccination at 12 years better than vaccination at other ages
Sponsor	NR	CDC*	CDC*	NR

*Not explicitly reported but inferred.

*WB*, World Bank; *NR*, Not Reported; *NA*, Not Applicable; *CDC*, US Centers for Disease Control and Prevention.

**Table 5:** Cost-benefit analyses of vaccination programs in the general population

**First author [Reference]**	**Bjerregaard [**[28]**]**	**Pelletier [**[29]**]**	**Elo [**[31]**]**	**Berger [**[30]**]**
Country	Denmark	Canada	Finland	Israel
Year	1991	1998	1979	1990
WB income group	High	High	High	High
Comparators	1. Vaccinate 15-month and 12-yr-olds	1. 1-dose child vaccination campaign	1. Vaccinate 13-yr-olds & post-partum women	1. Vaccinate children from 1 – 12 years
	2. Vaccinate only 15-month-olds	2. 2-dose child vaccination campaign	2. Vaccinate 13-yr-olds & 1-yr-olds	2. Vaccinate only 12-yr-olds (routine)
		3. 1-dose child vaccination	3. Vaccinate only 1-yr-olds over 20 years	
		4. 2-dose child vaccination		
Perspective	Societal*	Societal*	Societal*	NR
Cost components measured	OP visits; prescriptions; hospitalizations; vaccines	OP visits; hospitalizations; laboratory tests; nursing home care; special education; indirect costs (lost productivity for illness, disability and premature death)	Fetal loss; fetal damage; annual costs; long-term costs	Vaccine; vaccination side effects; serology; OP visits; hospitalizations; hearing aids; mothers’ work loss;
Method of cost estimation	Micro-costing	Micro-costing; Lifetime earnings (for indirect costs)	Top-down costing based on Delphi panel	Micro-costing
Method of benefits estimation	Averted costs	Averted costs	Averted costs	Averted costs
Time period for costs and benefits	20 years	Lifetime	30 years	13 years
Discounting (Rate)	NR	Yes (5%)	Yes (6%)	Yes (5 and 10%)
Results—Benefit-cost ratio	1. 3	1. 2.6	1. 10	1. 1.1
	2. 2	2. 2.9	2. 3	2. 1.8
		3. 3.6	3. 6	
		4. 4.3		
Stated conclusion	Vaccinating both age groups is preferable	The benefits of a second dose outweigh the costs	Vaccinating 13-yr-old girls and postpartum women was preferable	Vaccinating infants and adolescent girls is preferable
Sponsor	NR	LCDC	NR	NR

*Not explicitly reported but inferred.

*WB*, World Bank; *NR*, Not Reported; *NA*, Not Applicable; *OP*, Out Patient; *CDC*, US Centers for Disease Control and Prevention; *LCDC*, Canada Laboratory Center for Disease Control.

**Table 6:** Cost-benefit analyses of vaccination programs in the general population

**First author [Reference]**	**Golden [**[32]**]**	**Kommu [**[33]**]**	**Irons [**[34]**]**
Country	Israel	Barbados	Caribbean
Year	1984	1998	2000
WB income group	High	High	Upper-middle
Comparators	1. Vaccinate all 1 - 12-yr-olds	1. Rubella elimination initiative	1. Initiative to interrupt rubella transmission
	2. Vaccinate pubertal girls	2. None	2. None
	3. Vaccinate adult females		
Perspective	Societal*	Payer*	Payer*
Cost components measured	Laboratory tests; abortions; primary care; institutional care; lost work days;	NR	NR
Method of cost estimation	Micro-costing	NR	NR
Method of benefits estimation	Averted costs	NR	NR
Time period for costs and benefits	10 years	15 years	20 years
Discounting (Rate)	Yes (10%)	NR	NR
Results—Benefit-cost ratio	1. 1	1. 4.7	1. 13.3
	2. 2	2. –	2. –
	3. Negative		
Stated conclusion	Vaccination of children and pubertal girls is preferable	The rubella elimination program using MMR was cost-beneficial	The rubella elimination program using MMR was cost-beneficial
Sponsor	NR	NR	NR

*Not explicitly reported but inferred.

*WB*, World Bank; *NR*, Not Reported; *NA*, Not Applicable; *OP*, Out Patient; *CDC*, US Centers for Disease Control and Prevention; *LCDC*, Canada Laboratory Center for Disease Control.

Four studies [26,27,30,31] compared MMR with monovalent rubella vaccine. All but the study performed in 1979 in Finland [31] found that vaccination with MMR is preferable. White et al. [26] performed a cost-benefit analysis of a routine childhood MMR vaccine program in the US compared to no vaccination, or vaccination with individual (measles, mumps or rubella) antigens and found that the MMR vaccine had a higher benefit to cost ratio than the monovalent ones and would be preferred. Hatzandrieu and Halpern [27] replicated the methods of White et al. [26] with similar findings, that introducing MMR vaccine was more cost-beneficial than monovalent rubella vaccine. Berger [30] conducted an analysis in Israel of the costs and benefits of introducing a mumps-rubella vaccine to monovalent antigens and found that the combined vaccine was preferable. Elo [31] on the other hand, found that vaccination with rubella vaccine in Finland was more cost-beneficial.

Six studies compared immunization delivery strategies targeting different age groups. Three studies, one in Denmark [28] and two in Israel [30,32], found that it was more cost-beneficial to vaccinate infants and pubertal girls. One study [24] in the US found that it was better to vaccinate 12 year old girls than two year old children. The other study in Finland [31] found that it was better to vaccinate pubertal girls and postpartum women.

Two studies evaluated the costs and benefits of rubella elimination. Kommu and Chase [33] estimated a benefit to cost ratio of 4.7. Irons et al. (2000) [34] performed a cost-benefit analysis of rubella elimination in the English-speaking Caribbean. They estimated a disease burden of 1,500 cases in all the countries and an expenditure on CRS of (2012) US$ 126 million. A campaign to interrupt rubella transmission would cost (2012) US$9.1 million and result in a benefit to cost ratio of 13.3 for a rubella and CRS eradication campaign involving mass vaccination in 18 countries.

#### Cost-effectiveness analyses of rubella vaccination programs

Table 7 is a summary of the five cost-effectiveness studies that evaluated rubella vaccination. Of these, four were conducted in high-income countries (one each in France [35], Slovakia [37], the USA [38] and Netherlands [39]) and the other was conducted in a lower-middle-income country (Guyana) [36]. Three studies assessed the cost-effectiveness of national rubella or MMR vaccination [35,37,38], one study evaluated different approaches to screening women to identify candidates for immunization in the [39] and the other evaluated rubella elimination in Guyana [36]. The three studies of national-level vaccination found that it would be cost-effective. In Hudeckova’s study in Slovakia [37], the introduction of MMR vaccination yielded cost savings of (2012) $16 million) and a cost per case prevented of (2012) $313. Zhou’s study found that a two dose MMR program in the US would save (2012) $231 per rubella case prevented and (2012) $683,813 per CRS case prevented [38].

**Table 7:** Cost-effectiveness and cost-utility analyses of vaccination programs in the general population

**First author [Reference]**	**Chapalain [**[35]**]**	**Kandola [**[36]**]**	**Hudeckova [**[37]**]**	**Zhou [**[38]**]**	**Lugner [**[39]**]**
Country	France	Guyana	Slovakia	USA	Netherlands
Year	1978	1998	2001	2004	2010
WB income group	High	Upper-middle	High	High	High
Comparators	1. Vaccinate 13-yr-olds and women	1. Rubella eradication campaign	1. National vaccination campaign	1. Rubella vaccination program	1. Screen and vaccinate all unvaccinated in LVR
	2. No vaccination	2. No campaign	2. No campaign	2. No vaccination program	2. Screen and vaccinate all pregnant in LVR
					3. Screen and vaccinate all unvaccinated in NL
Perspective	Payer*	NR	Payer	Societal	Payer
Cost components measured	Vaccination; specialist training; research; antenatal supervision; improvement of obstetric care; intensive care	NR	NR**	Vaccination; OP care; hospitalization; institutional care; special care; Indirect (premature mortality, disability, missed work)	Vaccination; screening; healthcare costs
Method of cost estimation	Top-down costing	NR	NR**	Micro-costing; Human capital approach (indirect costs)	Micro-costing
Time period for costs and benefits	15 years	5 years	NR**	40 years	16 years
Discounting (Rate)	Yes (NR)	NR	NR**	Yes (3%)	Yes (4%)
Outcomes	Mortality; lives saved	CRS cases prevented	Rubella cases prevented	Cases prevented; lives saved	QALYs
Method of outcome measurement	Primary analysis of program data	NR	NR**	Decision model	Model-based
Results—ICER (2012 US$/Outcome)	$20,474/Life saved	$3,335/CRS case prevented	$313/Case prevented	Vaccination program was dominant	1 dominated 2; the ICER comparing 3 to 1 was $114,575/QALY gained
Stated conclusion	The immunization program was cost-effective	Rubella eradication is highly cost-effective	National MMR immunization program was cost-effective	Two-dose MMR vaccination program is cost-effective	Screening and vaccinating all unvaccinated women is the most cost-effective
QHES score	30	NS	NS	93	62
Sponsor	NR	NR	NR	CDC	NL CIDC

*Not explicitly reported but inferred **Article in Slovak.

*WB*, World Bank; *NR*, Not Reported; *NS*, Not Scored; *OP*, Out Patient; *LVR*, Low vaccination coverage regions; *NL*, Netherlands; *CDC*, US Centers for Disease Control and Prevention; *CIDC*, Center for Infectious Disease Control; *QALYs*, Quality-Adjusted Life-Years.

Lugner et al. [39], evaluated screening followed by vaccination of susceptible women. They compared the screening of non-vaccinated pregnant women in areas of low-vaccine coverage, the screening of all pregnant women in these areas, and the screening of all non-vaccinated pregnant women throughout the country. The study, which was performed from the perspective of the healthcare system, found that screening non-vaccinated women in areas with low vaccine coverage and vaccinating the susceptible was the most cost-effective with a cost/QALY of (2012) $2,300 compared to screening all women in areas of low vaccine coverage ((2012) $62,000/QALY) and screening all women in the country ((2012) $115,000/QALY).

Kandola [33] estimated that a rubella elimination campaign in Guyana would cost (2012) $950,000. The long-term financial savings would be US$ 36.9 million (undiscounted) for a cost-effectiveness ratio of (2012) $3,335 per CRS case prevented.

## Discussion

This review of published economic analyses of rubella, CRS, and rubella vaccination suggests some general themes about economics of the disease and the value of vaccination. In high- and middle-income countries, CRS is such a costly disease that rubella vaccination is a high value intervention.

The review revealed that a broad consensus over four decades and in a variety of high- and middle-income countries has existed, i.e., rubella vaccination (including different programmatic approaches to vaccination) is cost-effective or cost-beneficial. This has been true for vaccination of children of both genders (to interrupt rubella transmission) [24,25,28-33]; vaccination of teenage girls or adult women (to prevent CRS) [39,41]; and vaccination of children, adolescent girls, or adult women [22,24,25,28,30-32]. In general, vaccination to prevent CRS was more cost-beneficial or cost-effective than vaccination to interrupt rubella transmission.

With regard to testing and vaccination of hospital workers, earlier studies [17,18] suggested that blind vaccination was preferable to targeted vaccination (after serological screening). But more recent studies found that blind vaccination is only slightly more costly [20] or less costly than targeted vaccination [19]. Cost analyses also suggest that vaccination is affordable for health workers, [17,18] among children [21] and among postpartum women [23]. This finding suggests that as vaccine prices have fallen over the years, blind vaccination has become more favorable compared to targeted vaccination.

The use of mass campaigns was modeled in Canada [29] and the Caribbean [16,33,34,36] and in conjunction with routine immunization. One study evaluated the use of monovalent rubella vaccine [21] and another MR in an evaluation of adding childhood immunization to an already-existing program of immunizing 12-yr old girls [30]. The others used MMR only, [26,28,32,37,38] MMR and monovalent rubella vaccine [25,33,34,36] or MMR for routine and MR for campaigns [29]. Both campaigns in conjunction with routine immunization and routine programs, regardless of the vaccine presentation used, were cost-beneficial.

One of the most striking findings was that no studies have been performed in low-income countries where the highest burden of rubella and CRS exists. This may be due to the limited treatment options for CRS and use of RCV in these countries. However, now that the GAVI Alliance has introduced funding for RCV into its program [7], demand for these analyses should increase.

Conducting economic analysis of introducing RCV in low-income countries would provide useful information for program managers and policymakers on which service delivery strategies are cost-effective and cost-beneficial and which age groups should be targeted. Economic analyses on RCV will not be needed in every country before RCV since studies from countries with similar socio-demographic circumstances can be informative [42,43]. However, given the high cost of CRS and the low cost of vaccination, as well as increasing price inflation of healthcare and other services used in the care of disabled children, that the net economic benefit of rubella vaccination will remain substantial in analyses in this setting [44].

Economic analyses of RCV should examine, for example, whether efficiencies can be gained from using a combination vaccine (MMR) compared to monovalent vaccine [26]. Since measles vaccination is already part of the routine immunization program in all low-income countries [42], studies could assess the cost-effectiveness of substituting MMR and MR for monovalent measles vaccine. Plotkin [1] suggests that there will be a large opportunity cost if measles immunization activities do not take advantage of reducing rubella simultaneously and that the cost-effectiveness of rubella vaccination in low-income countries should be taken into account within the context of measles control and elimination.

There are two ways of implementing new rubella vaccine initiatives in countries: vaccinating adolescent girls and/or women of childbearing age to reduce CRS and vaccinating children using combined measles and rubella vaccines (MR or MMR) to interrupt rubella transmission altogether [42,43]. It would be worthwhile to perform economic analyses to determine which option should be implemented in low-income countries. Three factors make it likely that vaccination of teenage girls and women of childbearing age will be more efficient in low-income than in high-income countries: 1) later immunization reduces the time to benefits because there are fewer years to discount; 2) the costs of acute childhood rubella are so small that the net benefit of early vaccination is diminished; and 3) since the vaccine provides immunity for at least twenty years, immunity would increase among adults [44].

We also found some methodological issues related to discounting. Studies differed as to whether or not discounting was performed and which discount rate was used. Discounting allows analysts to value current costs more than future costs given the opportunity cost associated with current relative to future expenditure [45]. It is desirable to avert healthcare costs today because such savings can be invested for a future return [45]. Many of the older studies used discount rates as high as 5% [30] or 10% [26,30] although these still found that vaccination was associated with net economic benefits. Future studies should consider using lower discount rates since the choice of discount rate and number of years of discounting affects the potential cost-effectiveness of childhood vaccination.

None of the economic studies reviewed considered herd immunity or potential adverse outcomes associated with vaccination. When herd immunity is taken into account, it increases the attractiveness of mass vaccination campaigns at the onset of programs and on-going vaccination of children [44]. On the other hand, as mentioned earlier, if rubella coverage is too low, RCV could increase the rubella susceptibility of women. Future studies might consider using dynamic modeling methods as the basis of economic evaluations to capture the potential effects of coverage and herd immunity.

Only one of the studies, a cost-effectiveness analysis [39], included adjustments for disability. Because rubella has potentially significant impacts on the quality of life of CRS patients and their caretakers, future studies might consider estimating the cost per DALY saved or cost per QALY gained.

Rubella has both maternal and child health consequences because maternal infection can cause abortion. Some studies measured the costs of abortions [32] but most did not. In low-income countries where the burden of rubella and CRS are high and induced abortions are illegal, many more babies with CRS may be born without the option of therapeutic abortion and this would have economic consequences. Moreover, models may need to consider levels of fertility and induced abortion as they relate to CRS burden, as well as the economic consequences of safe or unsafe abortions or abortions performed in response to a diagnosis of CRS. Since vaccination may prevent many medically-indicated abortions in developed countries and clandestine abortions in developing countries, studies might quantify this potential economic and health outcomes impact.

## Conclusions

Congenital rubella syndrome is costly and rubella vaccination programs, including the vaccination of health workers, children, and women has favorable cost-effectiveness, cost-utility, or cost-benefit ratios in high- and middle-income countries.

However additional studies are required to include low-income countries, tackle methodological limitations, establish the efficiency of immunization programs in conjunction with measles immunization, and establish the most efficient rubella vaccination schedule.

### Endnotes

^1^Barbados is rated as a high income country by the World Bank.

## Competing interests

The authors declare that they have no competing interests.

## Authors’ contributions

JBB and AL conceived of the study. JBB and IM performed the literature search and identified the articles for review. All the authors reviewed the articles. JBB prepared the first draft of the manuscript. IM and AL revised the manuscript. All authors have read and approved the final manuscript.

## Pre-publication history

The pre-publication history for this paper can be accessed here:

http://www.biomedcentral.com/1471-2458/13/406/prepub

## References

[B1] Plotkin SA Rubella eradication Vaccine 2001 19 25–26 3311 3319 11348695 10.1016/s0264-410x(01)00073-1

[B2] Cutts FT Vynnycky E Modelling the incidence of congenital rubella syndrome in developing countries Int J Epidemiol 1999 28 6 1176 1184 10.1093/ije/28.6.1176 10661666

[B3] Castillo-Solorzano C Marsigli C Bravo-Alcantara P Flannery B Ruiz Matus C Tambini G Gross-Galiano S Andrus JK Elimination of rubella and congenital rubella syndrome in the Americas J Infect Dis 2011 204 2 S571 S578 10.1093/infdis/jir472 21954249

[B4] Reef SE Strebel P Dabbagh A Gacic-Dobo M Cochi S Progress toward control of rubella and prevention of congenital rubella syndrome–worldwide J Infect Dis 2009 1 204 S24 S27 204 Suppl 10.1093/infdis/jir155 21666168

[B5] World Health Organization Rubella vaccines. WHO position paper Wkly Epidemiol Rec 2000 75 161 172

[B6] World Health Organization Rubella vaccines. WHO position paper Vaccine 2011 29 48 8767 8768 10.1016/j.vaccine.2011.08.061 21930175

[B7] GAVI Alliance 2013 http://www.gavialliance.org/support/nvs/rubella

[B8] Hinman AR Irons B Lewis M Kandola K Economic analyses of rubella and rubella vaccines: a global review Bull World Health Organ 2002 80 4 264 270 12075361 PMC2567775

[B9] Moher D Liberati A Tetzlaff J Altman DG Preferred reporting items for systematic reviews and meta-analyses: the PRISMA statement PLoS Med 2009 6 7 e1000097 10.1371/journal.pmed.1000097 19621072 PMC2707599

[B10] World Bank Data How we classify countries 2013 http://data.worldbank.org/about/country-classifications

[B11] Chiou CF Hay JW Wallace JF Bloom BS Neumann PJ Sullivan SD Yu HT Keeler EB Henning JM Ofman JJ Development and validation of a grading system for the quality of cost-effectiveness studies Med Care 2003 41 1 32 44 10.1097/00005650-200301000-00007 12544542

[B12] Ofman JJ Sullivan SD Neumann PJ Chiou CF Henning JM Wade SW Hay JW Examining the value and quality of health economic analyses: implications of utilizing the QHES J Manag Care Pharm 2003 9 1 53 61 14613362 10.18553/jmcp.2003.9.1.53 PMC10437166

[B13] Al-Awaidy S Griffiths UK Nwar HM Bawikar S Al-Aisiri MS Khandekar R Mohammad AJ Robertson SE Costs of congenital rubella syndrome (CRS) in Oman: evidence based on long-term follow-up of 43 children Vaccine 2006 24 40–41 6437 6445 16814433 10.1016/j.vaccine.2006.05.089

[B14] Lanzieri TM Parise MS Siqueira MM Fortaleza BM Segatto TC Prevots DR Incidence, clinical features and estimated costs of congenital rubella syndrome after a large rubella outbreak in Recife, Brazil, 1999–2000 Pediatr Infect Dis J 2004 23 12 1116 1122 15626948

[B15] Saad de Owens C Tristan de Espino R Rubella in Panama: still a problem Pediatr Infect Dis J 1989 8 2 110 115 2704601

[B16] Robinson CRS unit costing Final Report, Fourteenth Meeting of the English-speaking Caribbean EPI Managers, Castries, Saint Lucia, 18–20 November 1997 1998 Pan American Health Organization: Washington (DC)

[B17] Stover BH Adams G Kuebler CA Cost KM Rabalais GP Measles-mumps-rubella immunization of susceptible hospital employees during a community measles outbreak: cost-effectiveness and protective efficacy Infect Control Hosp Epidemiol 1994 15 1 18 21 10.1086/646812 8133004

[B18] Ferson MJ Robertson PW Whybin LR Cost effectiveness of prevaccination screening of health care workers for immunity to measles, rubella and mumps Med J Aust 1994 160 8 478 482 8170422

[B19] Celikbas A Ergonul O Aksaray S Tuygun N Esener H Tanir G Eren S Baykam N Guvener E Dokuzoguz B Measles, rubella, mumps, and varicella seroprevalence among health care workers in Turkey: is prevaccination screening cost-effective? Am J Infect Control 2006 34 9 583 587 10.1016/j.ajic.2006.04.213 17097453

[B20] Alp E Cevahir F Gokahmetoglu S Demiraslan H Doganay M Prevaccination screening of health-care workers for immunity to measles, rubella, mumps, and varicella in a developing country: What do we save? J Infect Public Health 2012 5 2 127 132 10.1016/j.jiph.2011.11.003 22541258

[B21] Fontanesi J De Guire M Kopald D Holcomb K The price of prevention. cost of recommended activities to improve immunizations Am J Prev Med 2004 26 1 41 45 10.1016/j.amepre.2003.09.009 14700711

[B22] Gudnadottir M Cost-effectiveness of different strategies for prevention of congenital rubella infection: a practical example from Iceland Rev Infect Dis 1985 7 Suppl 1 S200 S209 3923594 10.1093/clinids/7.supplement_1.s200

[B23] Haas DM Flowers CA Congdon CL Rubella, rubeola, and mumps in pregnant women: susceptibilities and strategies for testing and vaccinating Obstet Gynecol 2005 106 2 295 300 10.1097/01.AOG.0000171110.49973.e3 16055578

[B24] Schoenbaum SC Hyde JNJBL Crampton K Benefit-cost analysis of rubella vaccination policy N Engl J Med 1976 294 6 306 310 10.1056/NEJM197602052940604 813141

[B25] Stray-Pedersen B Economic evaluation of different vaccination programmes to prevent congenital rubella NIPH Ann 1982 5 2 69 83 6820478

[B26] White CC Koplan JP Orenstein WA Benefits, risks and costs of immunization for measles, mumps and rubella Am J Public Health 1985 75 7 739 744 10.2105/AJPH.75.7.739 3923849 PMC1646302

[B27] Hatziandreu EJ BR Halpern RE A Cost-Benefit Analysis of Measles-Mumps-Rubella (MMR) Vaccine. Final Report Prepared for the National Immunization Program, Centers for Disease Control and Prevention, USA, 13th May 1994 1994

[B28] Bjerregaard P Economic analysis of immunization programmes Scand J Soc Med Suppl 1991 46 115 119 1805361

[B29] Pelletier L Chung P Duclos P Manga P Scott J A benefit-cost analysis of two-dose measles immunization in Canada Vaccine 1998 16 9–10 989 996 9682349 10.1016/s0264-410x(97)00281-8

[B30] Berger SA Ginsberg GM Slater PE Cost-benefit analysis of routine mumps and rubella vaccination for Israeli infants Isr J Med Sci 1990 26 2 74 80 2108102

[B31] Elo O Cost-benefit studies of vaccinations in Finland. Developments in biological standardization Dev Biol Stand 1979 43 419 428 118069

[B32] Golden M Shapiro GL Cost-benefit analysis of alternative programs of vaccination against rubella in Israel Public Health 1984 98 3 179 190 10.1016/S0033-3506(84)80043-8 6429710

[B33] Kommu Follow-up of rubella issues and costing of CRS in Barbados Final Report, Fourteenth Meeting of the English-speaking Caribbean EPI Managers, Castries, Saint Lucia, 18–20 November 1997 1998 Washington (DC): Pan American Health Organization http://hist.library.paho.org/english/epi_mana.pdf

[B34] Irons B Lewis MJ Dahl-Regis M Castillo-Solorzano C Carrasco PA de Quadros CA Strategies to eradicate rubella in the English-speaking Caribbean Am J Publ Health 2000 90 10 1545 1549 10.2105/ajph.90.10.1545 PMC1446383 11029986

[B35] Chapalain MT Perinatality: French cost-benefit studies and decisions on handicap and prevention CIBA Found Symp 1978 59 193 206 152699 10.1002/9780470720417.ch11

[B36] Kandola CRS cost burden analysis for Guyana Final Report, Fourteenth Meeting of the English-speaking Caribbean EPI Managers, Castries, Saint Lucia, 18–20 November 1997 1998 Washington (DC): Pan American Health Organization http://hist.library.paho.org/english/epi_mana.pdf

[B37] Hudeckova H Straka S Avdicova M Rusnakova S Health and economic benefits of mandatory regular vaccination in Slovakia. IV. Measles, rubella and mumps Epidemiol Mikrobiol Imunol 2001 50 1 31 35 11233671

[B38] Zhou F Reef S Massoudi M Papania MJ Yusuf HR Bardenheier B Zimmerman L McCauley MM An economic analysis of the current universal 2-dose measles-mumps-rubella vaccination program in the United States J Infect Dis 2004 189 Suppl 1 S131 S145 15106102 10.1086/378987

[B39] Lugner AK Mollema L Ruijs WL Hahne SJ A cost-utility analysis of antenatal screening to prevent congenital rubella syndrome Epidemiol Infect 1172 138 8 1172 1184 20018128 10.1017/S0950268809991336

[B40] Historical consumer price indices (1969 to 2012) for baseline countries/regions International Financial Statistics, International Monetary Fund https://www.google.com/url?sa=t&rct=j&q=&esrc=s&source=web&cd=1&ved=0CDAQFjAA&url=http%3A%2F%2Fwww.ers.usda.gov%2Fdatafiles%2FInternational_Macroeconomic_Data%2FHistorical_Data_Files%2FHistoricalCPIsValues.xls&ei=U6mCUar4Fqe7iwLwjICwBw&usg=AFQjCNFa-CAxLV5VSHwvI3ePYTffQ-lCdQ&sig2=j_OgSEja8QTrfohDH2QPfg&bvm=bv.45960087,d.cGE

[B41] Chapalain MT Perinatality: French cost-benefit studies and decisions on handicap and prevention Ciba Foundation Symposia 1978 59 193 206 152699 10.1002/9780470720417.ch11

[B42] World Health Organization Rubella vaccines. WHO position paper Wkly Epidemiol Rec 2011 86 301 316 72 21766537

[B43] Who P Rubella vaccines: WHO position paper–recommendations Vaccine 2011 29 48 8767 8768 10.1016/j.vaccine.2011.08.061 21930175

[B44] Schoenbaum SC Benefit-cost aspects of rubella immunization Rev Infect Dis 1985 7 Suppl 1 S210 S211 3923595 10.1093/clinids/7.supplement_1.s210

[B45] Torgerson DJ Raftery J Economic notes. Discounting BMJ 1999 319 7214 914 915 10506056 10.1136/bmj.319.7214.914 PMC1116731

